# Differences in perceived parental practices across attachment styles in adult children of alcoholic fathers

**DOI:** 10.1016/j.heliyon.2022.e10317

**Published:** 2022-08-19

**Authors:** Ivan Suneel, Elizabeth Schwaiger, Syeda Saniya Zehra, Abia Nazim

**Affiliations:** Department of Psychology, Forman Christian College (A Chartered University), Pakistan

**Keywords:** Attachment, Parental practices, Adult children of alcoholics, Alcoholism

## Abstract

**Background:**

Alcoholism is a growing problem in Pakistan. Problems related to alcoholism affects the family members especially children of alcoholics who are likely to have biased perceptions of parental practices which in turn affects their attachment patterns.

**Objective:**

To analyze the differences in perceptions of parental practices across three attachment styles namely avoidant, anxious/ambivalent, and secure attachments of the adults who are children of alcoholic parents.

**Method:**

The study is a correlational research design with a sample of 330 participants selected through purposive sampling technique. The participants were adults who are children of alcoholics from nuclear family systems, whose fathers were admitted in a rehabilitation center for the treatment of alcoholism for the second time.

**Results:**

MANOVA computed to examine differences in perception of parental practices across attachment styles was significant [*F*(12, 632) = 53.130, *p* < .001, Pillai's Trace = 1.003, partial eta squared = .501], indicating that those with different attachment styles perceive parental overprotectiveness, emotional warmth, and rejection differently.

**Conclusion:**

Perceptions about parental practices for father can be linked with alcoholism, while for mother the perceptions about parental practices are a product of attachment mainly.

**Implications:**

The findings can be used to devise counseling and therapeutic plans for adults who are children of alcoholics and help in educating them about the condition of alcoholism.

## 1 Introduction

In accordance with the religious context of Pakistan, the consumption of drugs like alcohol is banned (Haviland, 2013). Pakistan battles growing alcohol addiction and the drug is only available through illegal means (Haviland, 2013). The practice of consumption of alcohol is a taboo in the society, however its prevalence can still be witnessed among the citizens. Despite the ban, an estimated 44.9% of adolescents from the schools of Gilgit-Baltistan reported the lifetime use of alcohol while 22.6% are under serious risk from the substance abuse (Shahzad et al., 2019; Hussain, 2017). Another survey conducted at a hospital's OPD ward in Abbottabad showed that one out of 10^th^ patients seeking medical services report the use of alcohol, that is 63% (Subhan et al., 2020). Alcoholism greatly affects communities and different domains of an individual's life especially family (Subhan et al., 2020) where the members especially children of alcoholic parent (COA) suffer greatly in terms of stressors (Hussong et al., 2008) and general mental well-being (Subhan et al., 2020).

Research have shown that the parent-child relationships are usually strained when the normal course of parental related interactions with the children is disrupted, such as in the case of parental alcoholism (Mahato et al., 2009) The parental rearing practices can greatly affect the parent-child relationship in terms of development of the individual (Maccoby, 2000) thereby influencing the attachment relationship with primary caregivers. The ethological theory of attachment proposes that the need for attachment is innate and has a survival value in terms of how these attachments can serve as basis for attachments later in life (Bowlby, 1969/1982). The response of primary caregivers, that is how they interact with their children, usually from the biological parents, determines the nature of attachment (i.e., secure, insecure). The parental practices can be regarded as the responses which direct the course of attachment with these primary caregivers. These early childhood attachments result in internal working models of self and others which are a blueprint for future relationships (Bowlby, 1969/1982; Schneider, 1991).

The involvement of father especially during the developmental years of a child is of critical importance as it impacts the future experience of the children. Parents serve as role models for children and relationships with them help form the blueprint for future relationships (Bhamani, 2017). In the Pakistani collectivist culture where patriarchy is a dominant feature, the relationship with father is also likely to affect the life of children despite their attachments residing with other primary caregivers which is more often the mother (Suneel et al., 2020). This can be explained through the role of father being the breadwinner of the family who not only looks after the needs of the family but also has a decisional role in the family.

### 1.1 Alcoholism in Pakistan

Despite the bans on consumption of alcohol and the negative cultural and religious connotations attached to usage of alcohol, it is still being consumed at wide in many different regions of Pakistan (Haider and Chaudhry, 2008). Alcoholism can be referred to as the extensive use and dependency on alcohol where its consumption interferes with an individual's physical and psychological health and also causes problems in regards to his/her family and social life (Abbasi et al., 2014). A research conducted in one of the largest cities of Pakistan, Karachi indicated that among other types of drugs consumed by the local population, like cocaine (19%), crack-cocaine (15%), cannabis (5%) and so on, alcohol accounted for 5% of the sample population. A few reasons to mention given by the participants for using the drugs were, poor marital relations (21.4%), and poor paternal relations (15.8%; Ali et al., 2011). The population that consumes alcoholism is alarming in Pakistan, that is over a million (Haviland, 2013). According to Ghazal's (2015) research on the pattern of alcohol consumption, approximately 10 million people in Pakistan use alcohol, while 1 million out of these reportedly face alcohol related problems. One of the factors described in this research for the consumption of alcohol is due to stress (Ghazal, 2015).

### 1.2 Children of alcoholic parents (COA's/ACOA's)

The term adult children of alcoholics, refers to the adult population, whose parents are alcoholic, thus they are being referred to as adult children. Children of alcoholics (COA) face stressors in different domains of life, especially for family stressors domain which has a greater risk of developing as compared to their peers (Hussong et al., 2008). The environment of these COA's makes them vulnerable to a number of different psychological disorders and difficulties, for instance the COA's have increased rates of recurrent MDD (Vidal et al., 2012), as well as have comparatively higher risk of developing mental and behavioral disorders in regards to both maternal and paternal alcoholism (Raitasalo et al., 2018). These adult children of alcoholics (ACOA) have also reportedly experienced a role reversal in the parent-child relationship referred as “parentification” which places the responsibilities on these adults for looking after the emotional troubles of their respective members of the family (Kelley et al., 2007). COA's are at a greater risk for development of many psychological disorders like depression, attention deficit disorder (Díaz et al., 2007), along with unhealthy lifestyles like eating habits and lack physical activity (Serec et al., 2012). Substance abuse and related disorders are a common practice among these adolescents where the motivation for consumption is usually coping (Chalder et al., 2005; Serec et al., 2012). In these cases, parental alcoholism is regarded as an environmental factor which makes these adolescents vulnerable to consumption of alcohol and related problems (Steinhausen, 1995).

### 1.3 Attachment

Attachment theory is a collective work of John Bowlby and Mary Ainsworth. Bowlby presented the basis for the theory which was primarily focused on a child's ties with his/her mother and how that tie was affected when the child was separated from the mothers, where mothers were regarded as the primary caregivers (Bretherton, 1992). According to Bowlby and Ainsworth humans have an innate proneness towards seeking proximity with specific adults since infancy (Rajecki et al., 1978). As Bowlby suggested, attachment serves the purpose of providing an infant with safety, along with becoming a secure base which allows for exploring the environment (Davies, 2011). The ethological theory of attachment is based on four stages where the third stage is labeled as “separation anxiety” and is crucial in determining the attachment bond that an infant shares with a caregiver by identifying them as the primary caregivers (Berk, 2005).

To test Bowlby's attachment theory, Ainsworth (1985) came with the “Strange Situation” experiment where the infants were briefly separated from their primary caregivers, which induced anxiety. Two patterns of attachment were explained through this experiment in regards to how the infants responded to the stress of being separated from their caregivers, “securely attached” infants actively explored the environment when their mothers were present, while for the “insecure attachment” two further categorizations were made, “avoidant attachment pattern” “resistant or ambivalent attachment pattern” and “disorganized or disoriented” (Berk, 2005).

John Bowlby (1973) introduced the concept of “internal working models” which children develop on two levels, on the basis of the mechanics of their attachment relationship. The working of others is a blueprint of the relationship that a child had with his/her attachment figure during early childhood. The working model of self is where the child looks at him/herself in regard to relation with others that is how they were responded to in their attachment to primary caregivers. This working model of self is crucial for determining a child's self-representation and how they look at their abilities in the context of relationships (Davies, 2011).

### 1.4 Parental alcoholism and attachment

Research has shown that in families where both the parents are alcoholics, infants show greater insecure attachment patterns towards both, mother and father. The attachment patterns that emerged in the two groups, infants with alcoholic parents, and infants with non-alcoholic parents showed a marked difference. An estimate of 30% of infants with alcoholic parents exhibited disorganized attachment pattern towards their mothers in comparison to 5% for the non-alcoholic parents group. In comparison, around 40% of infants with alcoholic parents displayed avoidant attachment pattern towards their fathers while for the non-alcoholic parents group it was only accounted for 9% (Eiden et al., 2002).

It has been observed that families with history of alcoholism, that is adults with alcoholic biological fathers, were more likely to exhibit insecure attachment patterns, with fearful-avoidant and dismissed-avoidant attachment styles. In comparison to the participants who did not have a family history of alcoholism. Keeping hereditary aside, the insecure attachment patterns can put individuals in the high-risk category for development of Alcohol use disorders (Vungkhanching et al., 2004).

Bowlby's (1973) internal working models play an integral role in determining how an individual views the attachment figure and their own self. Research have shown that daughters of alcoholic fathers exhibited more insecure patterns of attachment in comparison to daughters of non-alcoholic fathers (Jaeger et al., 2000; El-Guebaly et al., 1993), as alcoholic parents are likely to be absent or unresponsive towards the child that leads to insecure attachment. However not all adult daughters of alcoholic fathers' experienced insecure attachment, this finding can be linked to the age of the participant at which the parent started consuming alcohol.

### 1.5 Parental practices

Parent-child relationship plays an important role in shaping an individual's life to an extent that parental-rearing behaviors are likely to influence the psychological well-being and development of the child (Maccoby, 2000). Research on school going children have shown that anxious parental-rearing behaviors has a significant positive relation with “anxiety disorders symptomology” such as generalized anxiety disorder and separation anxiety disorder (Muris and Merckelbach, 1998).

Research employing EMBU-C (Castro et al., 1993; as cited in Muris et al., 2000) have shown that for parental-rearing behaviors, an increased level of rejection, control and anxious rearing, along with a decreased level of emotional warmth are associated with higher levels of worry among children.

The rearing practices that parents indulge in greatly influence the mental health of their children and how they alter the course of lives based upon their perceptions about their parents. Children who belong to families which are different from the norm, perceive and attribute the traits to their parents which point towards the parents being unloving or not very supportive (Bosco et al., 2003). A research on criminals was done, where five different offenses were considered such as “first degree murder, drug-trafficking, kidnapping”. The results of the research indicated that the type of offense to some degree can be linked to the parental practices such as “parental rejection, over protection” that individuals experienced while growing up, and how they can be attributed to the criminal behavior (Khan and Munaf, 2017). Parental bonding reportedly has a great impact on adult psychopathy, where factors such as “low maternal care” along with “low paternal overprotection” significantly contribute to psychopathy. These paternal practices are seen as disinterest of parents in children's life which has adverse effects such as the link between “low paternal overprotection” that is lack of supervision from parents with substance abuse disorders (Gao et al., 2009). The COA's are likely to experience “parentification” (Kelley et al., 2007) due to the unavailability of the alcoholic parent. They consider themselves as the caretakers of the alcoholic parent instead of being taken care of by the parent.

Previous research suggested a strong connection between parental alcoholism, attachment, and parental practices, but no research in Pakistan attempted to study this significant aspect of children of alcoholic parents. Therefore, the present research aims to explore if there is any difference in perception of parental practices across the three attachment styles.

## 2 Hypothesis

The three attachment styles, (i.e. secure, avoidant and anxious/ambivalent) will demonstrate different levels of perceptions of parenting practice of father and mother.

## 3 Method

### 3.1 Research design

A correlational research design was used to study the relationship between perceived parent practices and attachment. Through this research design the strength and magnitude of a relationship between the given variables can be studied.

### 3.2 Sampling strategy and sample

Non-probability sampling technique was employed to select the sample of current research as characteristics of the population were specialized and thus required careful selection. The sample consisted of 330 participants from 18 to 25 years of age with a mean of 21.4 (*SD*= 2.37). The sample size and the age range of the participants of this study were determined on the consideration of relevant literature.

#### 3.2.1 Inclusion criteria

The study consisted of participants whose fathers, for the second time, were admitted in a rehabilitation center for treatment of alcoholism. For the demographic requirements, these participants belonged to a nuclear family system with a minimum of 3 siblings including the participant. Furthermore, the minimum level of education of the participants required for this study was Matric.

#### 3.2.2 Exclusion criteria

The participants whose fathers were admitted in the rehabilitation center for the first time for the treatment of alcoholism were not selected. Individuals who reported their fathers to be abusing other drugs along with alcohol, were not considered for participation. Furthermore, those living in joint or broken family systems were not selected as study participants.

#### 3.2.3 Setting

The participants belonged to two major cities of Pakistan, Lahore and Islamabad, as these urbanized cities consisted of a number of rehabilitation centers operating in the private sector. There are mainly three kinds of drug treatment facilities in Pakistan, drug rehabilitation wards at the psychiatric wards of public hospitals, drug rehabilitation centers that operate in the private sector, and drug rehabilitation units run by the Non-Government Organizations (NGOs). The data was collected from December 2018 to January 2020. For current study, the data were only collected from the private rehabilitation centers that provide drug rehabilitation. The rationale for this was that family members are called in or have family counselling sessions more frequently in private rehabilitation centers, thus it was feasible to approach the family members and collect data. The selection of private rehabilitation center helped in employing a rather homogenous sample in terms of socio-economic status, level of education and way of living.

### 3.3 Measures

The instruments employed for this research were detailed demographic sheet, Attachment Questionnaire (Hazan and Shaver, 1987), and Egna Minnen Betraffende Uppfostran (Saleem et al., 2015).

#### 3.3.1 Demographic sheet

A detailed demographic sheet was specifically designed for the present study to record important personal information, such as gender, age.

#### 3.3.2 Attachment Questionnaire

In order to measure the adult attachment style of the study participants, Hazan and Shaver's (1987) adult attachment measure was used. It consisted of three subscales which measured the three attachment styles including secure, avoidant, and anxious/ambivalent attachment. An Urdu version of the scale was used for the present study which was reported to be psychometrically sound.

#### 3.3.3 Egna Minnen Betraffende Uppfostran -A

The Urdu version of Egna Minnen Betraffende Uppfostran (EMBU; Saleem et al., 2015) was used for assessing the perception of children for parental rearing practices for both the parents. The EMBU scale consisted of 27 items divided into 4 subscales, rejecting, emotional warmth, overprotective, and favoring. The responses on the scale were recorded on a 4-point Likert-type scale with values ranging from “never” to “always.” The internal consistency of the scale for both the parents was .83. While the Cronbach's Alpha for the current study for both father (*α* =.77) and mother (*α=* .86) were in the acceptable range.

### 3.4 Procedure

Research design and protocol was approved by research and ethical review board of the relevant institute. The aims and objectives of the present study were explained to the authorities of given rehabilitation centers in individual meetings. They were informed about the procedures to ensure the confidentiality and anonymity of the study participants. The data collection procedure was explained in detail with the assurance that the data collection would not hinder or disturb the routine schedules at the center and inviolacy would be avoided.

After seeking the formal permission from administration, researcher introduced himself to the participants, then explained the aims and purpose of the study. The participants were informed clearly that their participation in the study was voluntary and thus they had a choice to participate or decline. Furthermore, they were guided about the duration, that was 40 min, for completing the questionnaire. For the informed consent, the consent was verbal, as signing the informed consent triggered anxiety among the participants. The participants were then told that there were no right and wrong answers to any of the questions, instead they should mark the option which most adequately reflected their perception. Individual sessions were conducted to collect the data and all participants filled the questionnaires in the same order. Once the research questionnaires were completed, they were checked to see any missing items, which they were requested to fill. If the participants had still left them unfilled, then those forms were discarded.

### 3.5 Data analysis

The data were entered into SPSS software to analyze the results. Other than descriptive analyses, a multivariate analysis of variance (MANOVA) was computed to assess the primary hypothesis “there is a significant difference in perception of parental practices across the three attachment styles”.

## 4 Results

### 4.1 Descriptive statistics

The demographic information about the sample has been presented in Table 1. The findings of descriptive analyses of the study variables are presented in Table 2. Participants perceived their fathers being more rejecting of them as the highest mean score on EMBU subscales of father was on Father Rejection and compared to Mother Rejection this was significantly higher. On the other hand, participants perceived their mothers as emotionally warm as the highest mean score on EMBU mother scales is on Mother Emotional Warmth which was significantly higher compared to mean score on Father Emotional Warmth.

**Table 1:** Demographic information of the sample.

Variable	*F*	Percentage (%)
Gender Male Female	167 163	50.6 49.4
Education Matriculation Intermediate Graduation Post-graduation	55 122 108 45	16.7 37 32.7 13.6

**Table 2:** Descriptive statistics of the study variables.

Variable	*M (SD)*	*Minimum*	*Maximum*	*n (%)*
Attachment Style				
Secure				110 (33.3)
Avoidant				110 (33.3)
Anxious/Ambivalent				110 (33.3)
EMBU				
Father OP	11.07 (3.85)	6	23	
Father EW	9.87 (1.74)	9	31	
Father R	14.72 (5.87)	6	24	
Mother OP	10.86 (2.80)	6	18	
Mother EW	28.23 (4.89)	9	36	
Mother R	9.36 (3.68)	6	23	

*Note.* EMBU = Egna Minnen Betriifende Upfostron; OP = Overprotective; EW = Emotional Warmth; R = Rejecting.

### 4.2 Inferential statistics

#### 4.2.1 Pre-Analysis screening

The assumptions of sample size and power were met; however, several scales of the EMBU were transformed to improve normality (Father Overprotectiveness [Sqrt], Father Emotional Warmth [Inverse], and Father Rejection [Log10]). Six multivariate outliers were removed (Mahalanobis Distances, *p* < .001). Levene's test of Equality of Error Variances was significant (*p* < .001) therefore an alpha level of *p* = .01 was utilized for the univariate F-test (Tabachnick and Fidell, 2007). Assumptions regarding linearity, multicollinearity, and singularity were met (see Table 3 for correlations).

**Table 3:** Correlations among EMBU subscales.

Variable	1	2	3	4	5	6
1. EMBU F OP		.015	.654∗∗	.627∗∗	-.430∗∗	.441∗∗
2. EMBU F EW			.203∗∗	-.296∗∗	-.279∗∗	-.247∗∗
3. EMBU F R				.448∗∗	-.407∗∗	.716∗∗
4. EMBU M OP					-.248∗∗	.644∗∗
5. EMBU M EW						-.190∗∗
6. EMBU M R						

*Note.* EMBU = Egna Minnen Betriifende Upfostron; OP = Overprotective; EW = Emotional Warmth; R = Rejecting. ∗∗Correlation is significant at *p* < .01.

#### 4.2.2 MANOVA

To evaluate differences in perception of parental practices across attachment styles, MANOVA was performed comparing the three attachment styles (Secure, Avoidant, Anxious/Ambivalent) across the three levels of perception of parenting practices for both father and mother (six EMBU subscales). The MANOVA was significant [*F*(12, 632) = 53.130, *p* < .001, *Pillai's Trace* = 1.003, *partial eta squared* = .501] indicating a significant difference in perception of parental practices among the different attachment styles. The effect size was large (*partial eta squared* = .501).

When the results of the dependent variables were considered separately, all subscales except EMBU Father Emotional Warmth were significant (at the Bonferroni adjusted significance level of *p* < .008; see Table 4 for the results of Tests of Between Subjects Effects).

**Table 4:** Results of tests of between subjects effects.

Variable	*F (df)*	*p*	*Partial eta squared*
EMBU F OP	100.502 (2, 321)	<.001∗	.385
EMBU F EW	4.188 (2, 321)	.016	.025
EMBU F R	309.559 (2, 321)	<.001∗	.659
EMBU M OP	22.141 (2, 321)	<.001∗	.121
EMBU M EW	56.249 (2, 321)	<.001∗	.260
EMBU M R	83.886 (2, 321)	<.001∗	.343

*Note*. ∗Significant result based on Bonferroni adjusted significance level of p < .008.

**Father Subscales of the EMBU.** Examination of the marginal means of the individual father subscales of the perceived parental practices indicated that those with Avoidant attachment styles reported the lowest levels of Father Overprotectiveness, followed by Secure attachment style. Those with Anxious/Ambivalent attachment styles had the highest levels of perceived Father Overprotectiveness. For Father Rejection on the perceived parental practices inventory, the same pattern emerged: those with Avoidant attachment styles again had the lowest scores, followed by the Secure group and those with Anxious/Ambivalent attachment.

**Mother Subscales of the EMBU.** For Mother Overprotectiveness, once again, the same pattern emerged: those with Avoidant attachment scored lowest, followed by Secure and Anxious/Ambivalent. However, for Mother Emotional Warmth, the Anxious/Ambivalent group scored lowest, followed by the Avoidant and then Secure group. Finally, on the Rejection subscale, those with Secure attachment scored lowest, followed by the Avoidant attachment group. Those with Anxious/Ambivalent attachments scored the highest on this subscale.

## 5 Discussion and conclusion

Despite being an illegal substance, having publicly banned production, sale and consumption in Pakistan, alcohol is still massively used. The literature over the years has uncovered the popularity of alcohol in many regions of Pakistan and the related psychological problems that prevail because of its consumption. Clinicians have observed that families, especially children of these alcoholic parents are greatly impacted and have strained relationships with their respective parents in terms of their attachment style. According to the results of the current research, there were significant differences in perception of parenting practices among the three attachment styles, secure, anxious/ambivalent and avoidant attachment.

### 5.1 Father Overprotectiveness and father rejection

For the two subscales, Father Overprotectiveness, and Father Rejection, participants with avoidant attachment to father had the lowest scores followed by securely attached individuals in the middle, while those with anxious/ambivalent attachment scored the highest. One explanation may be that adult children with avoidant attachment do not consider their fathers to be overprotective in nature because these children learn to keep themselves at a distance from their alcoholic fathers in an attempt to protect themselves. From a very early age these children have learnt that the dynamics of the family do not allow them to make any emotional demands from their alcoholic fathers (Hall and Webster, 2007). They distance themselves in an attempt to protect themselves from the hostile behavior of the alcoholic parent when under influence of the drug (Tedgård et al., 2018).

Similarly, the scores for avoidant attachment on the father rejection subscale were the lowest which highlights the characteristics of avoidant attachment style (Ainsworth, 1985), where these adult children avoid the parental figure or caregiver or become indifferent about them which is why they do not consider the alcoholic father is rejecting because they never saw the father as accepting or responsible in the first place (Mahato et al., 2009). The family members of alcoholics use different defense mechanisms in order to deal with the challenges associated with the alcoholic family member (Jay, 2007). It is basically a defense that these adult children are using to protect their well-being indifferent since they do not see their father coming through for them, leading to a no strings attached relationship with the father

It may be that the lack of availability of the father in emotional and psychological capacity has led these individuals to become independent in terms of their role, or the role has reversed as the concept of ‘parentification’ (Kelley et al., 2007). Thus, the lack of emotional connectedness has led to a situation where no such bond with the alcoholic father was developed which suggested that the father would come through for the children leading to a no strings attached relationship with the father.

As for the securely attached ACOA's their scores depict that the children feel secure in their environment where the alcoholic father is present. This finding reflects that ACOA's do not see the father as overprotective or rejecting because they are indifferent as they are aware of the reality. These adult children probably do not look up to their fathers for his approval because they are self-reliant and have accepted the circumstances of their father being an alcoholic and have thus made their peace with it in order to keep the family functional by taking on roles as the ‘systemic theory’ (Bowen, 1974) suggests. These individuals have become independent in terms of their role, acknowledging the reality principle which makes them likely to be inclined towards adoption of role reversal referred to as ‘parentification’ (Kelley et al., 2007) because they are aware of the dysfunctional situation.

The scores on father's overprotectiveness and father rejection were the highest for anxious/ambivalent attachment. This suggests that on one hand side they see the father as overprotective, and on the flip side they see him as rejecting which indicates towards father's drinking habit and how that makes these individuals anxious towards the father because they witnessed an imbalance. On days when the father is sober, he may experience factors of guilt and look out for his children thereby exhibiting his overprotective side, as compared to when the father is under the influence of alcohol he shows his rejective side. These children are unaware and confused of how the alcoholic father would behave on a given instance due to the unpredictable behavior of the father. This inconsistent behavior leads to anxiousness and doubtfulness towards the caregiver which ties in with the principles of anxious/ambivalent attachment (Ainsworth, 1985).

### 5.2 Father Emotional Warmth

The results on this subscale were not significant for all three attachment styles, secure, avoidant, and anxious/ambivalent, showing that perhaps attachment styles with their fathers does not impact perception of parental practices as ACOA's do not depend on him for emotional warmth in the first place. Due to the father's alcoholism, this aspect has probably never been there and these ACOA's thus have experienced neglect and in some cases abuse from their alcoholic parent (Jose and Cherayi, 2020; Laslett et al., 2012). Issues of neglect and child abuse present in children with alcoholic parents are consistent with previous literature showing how parental substance abuse is related to child maltreatment and foster care placements (Smith et al., 2007). Furthermore, in many cases children observe paternal abuse in the context of domestic violence because the fathers are under the influence of drugs, research has shown such abusive parental relationships to be having co-morbidity with neglect and child abuse (Guterman and Lee, 2005) which can explain the lack of emotional warmth which was analyzed as per the current research.

Alcoholism especially in Muslim countries like Pakistan is a frowned upon act because as per Islam, consumption of alcohol is a sin (Michalak and Trocki, 2006). Research have shown that families with drug dependent members are seen in a rather stigmatizing manner by the society, as addicts and addiction is something which is looked down upon (Corrigan et al., 2006). This stands especially true for Pakistani societies which are fundamentally built around the principles of religion, Islam, which does not allow for such practices, and instead regards addiction as *“Haram”* that is forbidden (Mustafa and Makhdoom, 2021). Given the religious aspect, and social aspect drug consumption is generally frowned upon in Pakistani society, however the negative connotations for alcohol consumption still stands out to be a greater stigmatization in comparison to other drugs as it is not as common in public as for instance tobacco is (Nizami et al., 2011). This reasoning offers an explanation for lack of emotional warmth that the ACOA's reported because closeness to family member with alcoholism is disapproved and rather kept hidden due to its stigmatizing nature in the society.

### 5.3 Mother overprotectiveness

For Mother Overprotectiveness, the ones with avoidant attachment style scored the lowest, with secure attachment scores somewhere in between and the anxious/ambivalent attachment style having the highest scores.

Systemic theory suggests that family is a system with components which each contribute towards the smooth functioning of the family (Bowen, 1974). Systemic theory in regard to a household with an alcoholic member suggests that the equilibrium is disturbed because a member is out of the equation due to alcoholism. The mother in this case is likely to take care of the alcoholic spouse which can be regarded as an act of co-dependency. The mother is likely to get involved in this care taking role so to restore an equilibrium which portrays the image of the family as normal. This co-dependency leads to negative consequences for the family system from the perspective of alcoholism as the mother is unavailable for her children by tending to the needs of her alcoholic husband while other responsibilities like bonding with the children, are compromised (Bortolon et al., 2016). Another possible explanation perhaps for low scores on mothers’ overprotectiveness for avoidant attachment can be due to the nature of such style of attachment (Ainsworth, 1985) as the findings for avoidant attachment on the given subscale for father were also the same. It can thus be suggested that this lower overprotectiveness can be a parental perception that the adult children hold because of their attachment style, that is avoidant, rather than being a function of alcoholism.

### 5.4 Mother Emotional Warmth and Mother Rejection

The scores on the Mother Emotional Warmth subscale were the lowest for anxious/ambivalent attachment, followed by avoidant attachment, while the secure attachment style had highest scores on this subscale. On the subscale of Mother Rejection, the scores were lowest for secure attachment style, with avoidant attachment next, and then the highest scores were for anxious/ambivalent attachment.

The findings above for both Mother Emotional Warmth, and Mother Rejection subscales can be attributed to the characteristics of the different attachment styles (Ainsworth, 1985) and how this attachment pattern relates to the perceived parental practices in the case of the non-alcoholic parent (mother). For instance, secure attachment style and comparatively higher levels of Emotional Warmth along with low scores on Rejection subscales can be attributed to the trustworthy and reliant parent-child relationship. While on the contrary for anxious/ambivalent attachment the parent-child relationship is usually seen in terms of behaviors which portray the child feeling rejected or neglected (Berk, 2005).

The findings of present study provided some interesting insights, however further research can be done to study the link between attachment styles and perceived parental practices and how one of them might be bringing about a change in another.

Furthermore, from a therapeutic perspective, the research can be used for developing counseling techniques regarding children of alcoholics and designing more relevant psychoeducation material to help them better understand their parents’ alcoholism and support their recovery.

Moreover, the findings of this research can provide a better case for understanding the adult children of alcoholic parents to reduce their vulnerability towards alcoholism and other psychological disorders. Findings can also be helpful to conceptualize management plans to assist adult children of alcoholic parents to develop secure attachments with significant persons in their lives.

Lastly, literature could be developed to educate the masses about alcoholism as a condition which has biological reasons as well and its likely impact on the families of alcoholics, especially their children.

To conclude, differences in perception of parenting practices among the three attachment styles were noted. The perceptions about parenting practices for father can be attributed to alcoholism while for mother regarding emotional warmth and rejection subscales the perceptions are more likely to be a function of attachment than alcoholism.

## Declarations

### Author contribution statement

Ivan Suneel: Conceived and designed the experiments; Performed the experiments; Analyzed and interpreted the data; Contributed reagents, materials, analysis tools or data; Wrote the paper.

Elizabeth Schwaiger, Syeda Saniya Zehra, Abia Nazim: Conceived and designed the experiments; Analyzed and interpreted the data; Contributed reagents, materials, analysis tools or data; Wrote the paper.

### Funding statement

This research did not receive any specific grant from funding agencies in the public, commercial, or not-for-profit sectors.

### Data availability statement

Data will be made available on request.

### Declaration of interest’s statement

The authors declare no conflict of interest.

### Additional information

No additional information is available for this paper.

## References

[bib1] Abbasi M.H., Makhdoom P.A., Aaamir Y., Khan R.A., Mengal F.A., Habib H. (2014). Frequency of alcohol consumption cases among people of Quetta. J. Med. Health Sci..

[bib2] Ainsworth M.D. (1985). Attachments across the life span. Bull. N. Y. Acad. Med..

[bib3] Ali H., Bushra R., Aslam N. (2011). Profile of drug users in Karachi city Pakistan. Eas. Mediterr. Health J..

[bib4] Berk L.E. (2005).

[bib5] Bhamani S. (2017, May 21). Urban fathers' involvement in early childhood development: a case study from Pakistan. Perspectives.

[bib6] Bortolon C.B., Signor L., Moreira T.D., Figueiró L.R., Benchaya M.C., Machado C.A., Ferigolo M., Barros H.M. (2016). Family functioning and health issues associated with codependency in families of drug users. Ciência Saúde Coletiva.

[bib7] Bosco G.L., Renk K., Dinger T.M., Epstein M.K., Phares V. (2003). The connections between adolescents' perceptions of parents, parental psychological symptoms, and adolescent functioning. J. Appl. Dev. Psychol..

[bib8] Bowen M. (1974). Alcoholism as viewed through family systems theory and family psychotherapy. Ann. N. Y. Acad. Sci..

[bib9] Bowlby J. (1969/1982).

[bib10] Bowlby J. (1973).

[bib11] Bretherton I. (1992). The origins of attachment theory: John Bowlby and mary Ainsworth. Dev. Psychol..

[bib12] Chalder M., Elgar F.J., Bennett P. (2005). Drinking and motivations to drink among adolescent children of parents with alcohol problems. Alcohol Alcohol.

[bib13] Corrigan P.W., Watson A.C., Miller F.E. (2006). Blame, shame, and contamination: the impact of mental illness and drug dependence stigma on family members. J. Fam. Psychol..

[bib14] Davies D. (2011).

[bib15] Díaz R., Gual A., García M., Arnau J., Pascual F., Cañuelo B., Rubio G., Dios Y.D., Fernández-Eire M.C., Valdés R., Garbayo I. (2007). Children of alcoholics in Spain: from risk to pathology. Soc. Psychiatr. Psychiatr. Epidemiol..

[bib16] Eiden R.D., Edwards E.P., Leonard K.E. (2002). Mother–infant and father–infant attachment among alcoholic families. Dev. Psychopathol..

[bib17] El-Guebaly N., West M., Maticka-Tyndale E., Pool M. (1993). Attachment among adult children of alcoholics. Addiction.

[bib18] Gao Y., Raine A., Chan F., Venables P.H., Mednick S.A. (2009). Early maternal and paternal bonding, childhood physical abuse and adult psychopathic personality. Psychol. Med..

[bib19] Ghazal P. (2015). The growing problem of alcoholism in Pakistan: an overview of current situation and treatment options. Int. J. Endorsing Health Sci. Res..

[bib20] Guterman N.B., Lee Y. (2005). The role of fathers in risk for physical child abuse and neglect: possible pathways and unanswered questions. Child. Maltreat..

[bib21] Haider W., Chaudhry M.A. (2008). Prevalence of alcoholism in the Punjab, Pakistan. Biomedica.

[bib22] Hall C.W., Webster R.E. (2007). Risk factors among adult children of alcoholics. Int. J. Behav. Consult. Ther. (IJBCT).

[bib23] Haviland C. (2013).

[bib24] Hazan C., Shaver P. (1987). Romantic love conceptualized as an attachment process. J. Pers. Soc. Psychol..

[bib25] Hussain A. (2017, October 3). Drug abuse on the rise among youth in Gilgit Baltistan. News Lens Pakistan. http://www.newslens.pk/drug-abuse-on-the-rise-among-youth-in-gilgit-baltistan/.

[bib26] Hussong A.M., Bauer D.J., Huang W., Chassin L., Sher K.J., Zucker R.A. (2008). Characterizing the life stressors of children of alcoholic parents. J. Fam. Psychol..

[bib27] Jay D. (2007).

[bib28] Jaeger E., Hahn N.B., Weinraub M. (2000). Attachment in adult daughters of alcoholic fathers. Addiction.

[bib29] Jose J.P., Cherayi S.J. (2020). Effect of parental alcohol abuse severity and child abuse and neglect on child behavioural disorders in Kerala. Child Abuse Neglect.

[bib30] Kelley M.L., French A., Bountress K., Keefe H.A., Schroeder V., Steer K., Fals-Stewart W., Gumienny L. (2007). Parentification and family responsibility in the family of origin of adult children of alcoholics. Addict. Behav..

[bib31] Khan B., Munaf S. (2017). Difference in perceived childhood parenting among five criminal types. Bahria J. Prof. Psychol..

[bib32] Laslett A.-M., Room R., Dietze P., Ferris J. (2012). Alcohol’s involvement in recurrent child abuse and neglect cases. Addiction.

[bib33] Maccoby E.E. (2000). Parenting and its effects on children: on reading and misreading behavior genetics. Annu. Rev. Psychol..

[bib34] Mahato B., Ali A., Jahan M., Verma A.N., Singh A.R. (2009). Parent-child relationship in children of alcoholic and non-alcoholic parents. Ind. Psychiatr. J..

[bib35] Michalak L., Trocki K. (2006). Alcohol and Islam: an overview. Contemp. Drug Probl..

[bib36] Muris P., Meesters C., Merckelbach H., Hülsenbeck P. (2000). Worry in children is related to perceived parental rearing and attachment. Behav. Res. Ther..

[bib37] Muris P., Merckelbach H. (1998). Perceived parental rearing behaviour and anxiety disorders symptoms in normal children. Pers. Indiv. Differ..

[bib38] Mustafa A.B., Makhdoom B.J. (2021). Drug addiction in Pakistan: a step towards alleviation of sufferings. J. Sheikh Zayed Medi. Coll..

[bib39] Nizami S., Sobani Z., Raza E., Baloch N., Khan J. (2011). Causes of smoking in Pakistan: an analysis of social factors. JPMA (J. Pak. Med. Assoc.).

[bib40] Raitasalo K., Holmila M., Jääskeläinen M., Santalahti P. (2018). The effect of the severity of parental alcohol abuse on mental and behavioural disorders in children. Eur. Child Adolesc. Psychiatr..

[bib41] Rajecki D.W., Lamb M.E., Obmascher P. (1978). Toward a general theory of infantile attachment: a comparative review of aspects of the social bond. Behav. Brain Sci..

[bib42] Saleem S., Mahmood Z., Subhan S. (2015). Perceived parental practices and mental health problems: cross-cultural validation of EMBU-C on Pakistani adolescents. FWU J. Soc. Sci..

[bib43] Schneider E.L. (1991). Attachment theory and research: review of the literature. Clin. Soc. Work. J..

[bib44] Serec M., Švab I., Kolšek M., Švab V., Moesgen D., Klein M. (2012). Health-related lifestyle, physical and mental health in children of alcoholic parents. Drug Alcohol Rev..

[bib45] Shahzad S., Kliewer W., Ali M., Begum N. (2019). Different risk factors are associated with alcohol use versus problematic use in male Pakistani adolescents. Int. J. Psychol..

[bib46] Smith D.K., Johnson A.B., Pears K.C., Fisher P.A., DeGarmo D.S. (2007). Child maltreatment and foster care: unpacking the effects of prenatal and postnatal parental substance use. Child. Maltreat..

[bib47] Steinhausen H.-C. (1995). Children of alcoholic parents. A review. Eur. Child Adolesc. Psychiatr..

[bib48] Subhan F., Alam A., Ahmad I., Afridi A., Arshid A., Ahmad Z., Rauf K. (2020). Physician’s perspective on alcohol use among patients of tertiary care hospital Abbottabad Pakistan. Ann. Pharmacol. and Pharm..

[bib49] Suneel I., Saleem S., Mahmood Z. (2020). Mental health functioning of adult children of alcoholic fathers in Pakistan. Rawal Med. J..

[bib50] Tabachnick B.G., Fidell L.S. (2007).

[bib51] Tedgård E., Råstam M., Wirtberg I. (2018). An upbringing with substance-abusing parents: experiences of parentification and dysfunctional communication. Nord. Stud. Alcohol and Drugs.

[bib52] Vidal S.I., Vandeleur C., Rothen S., Gholam-Rezaee M., Castelao E., Halfon O., Aubury J., Ferrero F., Preisig M. (2012). Risk of mental disorders in children of parents with alcohol or heroin dependence: a controlled high-risk study. Eur. Addiction Res..

[bib53] Vungkhanching M., Sher K.J., Jackson K.M., Parra G.R. (2004). Relation of attachment style to family history of alcoholism and alcohol use disorders in early adulthood. Drug Alcohol Depend..

